# Link Between Metabolic Syndrome, Inflammation, and Eye Diseases

**DOI:** 10.3390/ijms26052174

**Published:** 2025-02-28

**Authors:** Kamila Pieńczykowska, Anna Bryl, Małgorzata Mrugacz

**Affiliations:** 1 Doctoral School, Medical University of Bialystok, ul. Jana Kilińskiego 1, 15-089 Bialystok, Poland; mynameiskama@gmail.com; 2 Department of Ophthalmology and Eye Rehabilitation, Medical University of Bialystok, 15-089 Bialystok, Poland; anna.bryl@umb.edu.pl

**Keywords:** metabolic syndrome, dry eye, diabetic retinopathy, glaucoma, age-related macular degeneration, inflammation

## Abstract

Metabolic syndrome (MetS)—a cluster of conditions including obesity, hypertension, dyslipidemia, and insulin resistance—is increasingly recognized as a key risk factor for the development of various eye diseases. The metabolic dysfunctions associated with this syndrome contribute to vascular and neurodegenerative damage within the eye, influencing disease onset and progression. Understanding these links highlights the importance of early diagnosis and management of metabolic syndrome to prevent vision loss and improve ocular health outcomes. This review explores the intricate interplay between metabolic syndrome, chronic low-grade inflammation, and eye diseases such as diabetic retinopathy, age-related macular degeneration, glaucoma, and dry eye syndrome. It highlights how inflammatory mediators, oxidative damage, and metabolic dysregulation converge to compromise ocular structures, including the retina, optic nerve, and ocular surface. We discuss the molecular and cellular mechanisms underpinning these associations and examine evidence from clinical and experimental studies. Given the rising global prevalence of metabolic syndrome, addressing this connection is crucial for improving overall patient outcomes and quality of life. Future research should focus on delineating the precise mechanisms linking these diseases as well as exploring targeted interventions that address both metabolic and ocular health.

## 1. Introduction

**Figure 1:**
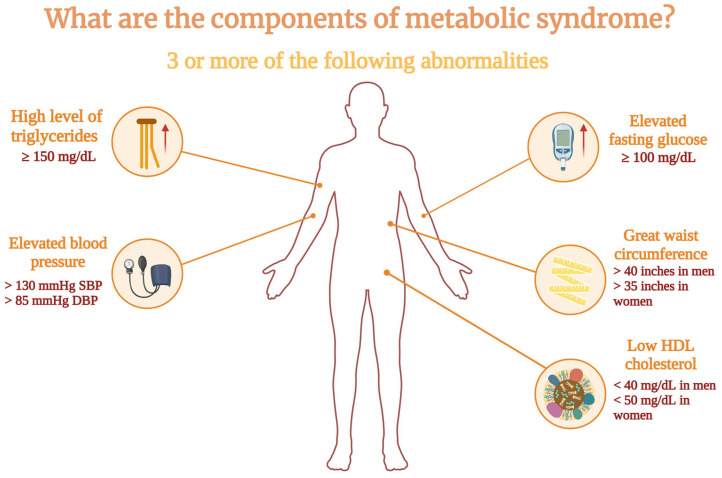
Components of metabolic syndrome. Created in BioRender. Pieńczykowska, K. (accessed on 20 February 2025).

Metabolic syndrome (MetS), defined by a cluster of metabolic issues such as central obesity, insulin resistance, hypertension, and dyslipidemia, significantly increases the risk of developing atherosclerotic cardiovascular diseases and type II diabetes mellitus. Worryingly, the prevalence of metabolic syndrome has risen sharply in recent decades, mirroring the global increase in obesity rates, and now affects over 20% of the populations in both America and Europe [1]. Based on the meta-analysis conducted on global data by Noubiap et al., the highest prevalence of metabolic syndrome was recorded in the Eastern Mediterranean Region (36.6%), while the lowest was in the African Region (23.1%). The worldwide prevalence of MetS also rose significantly as income levels increased [2]. The diagnosis of metabolic syndrome is based on the presence of three or more of the following metabolic abnormalities (also shown in Figure 1):

- Waist circumference greater than 40 inches (102 cm) in men and 35 inches (88 cm) in women;
- Serum triglycerides (TG) of 150 mg/dL or higher;
- Low high-density lipoprotein (HDL) cholesterol, below 40 mg/dL in men or below 50 mg/dL in women;
- Elevated fasting glucose of 100 mg/dL or more;
- Blood pressure (BP) readings of 130 mm Hg or higher systolic or 85 mm Hg or higher diastolic [3].

Between 2011 and 2016, the overall prevalence of metabolic syndrome rose slightly, from 32.5% to 36.9%, with notable increases among specific groups: women (from 31.7% to 36.6%), adults aged 20 to 39 (from 16.2% to 21.3%), Asian adults (from 19.9% to 26.2%), and Hispanic adults (from 32.9% to 40.4%) While rates of metabolic syndrome stay similar between men and women, they increase with age, affecting about one in five young adults and nearly half of those over 60. Among Hispanic adults over 60, almost 60% have metabolic syndrome [4]. Metabolic syndrome is no longer limited to adults; it also affects children and adolescents. In 2020, it was reported by Noubiap et al., that 3% of children and 5% of adolescents worldwide had this condition. The highest prevalence of metabolic syndrome in children occurred in Nicaragua (5.2%, range: 2.8–10.4), Iran (8.8%, range: 8.0–9.6), and Mexico (12.3%, range: 11.0–13.7). Among adolescents, the highest prevalence estimates were observed in Iran (9.0%, range: 8.4–9.7), the United Arab Emirates (9.8%, range: 8.5–10.3), and Spain (9.9%, range: 9.1–10.8) [5]. Socioeconomic inequalities may elevate the risk of developing metabolic syndrome, particularly among women [6]. On the other hand, a study conducted in Iran by Niknam et al. revealed higher prevalence of metabolic syndrome and its components in men [7]. Moreover, there seems to be a link between persistently high metabolic syndrome scores and an increased risk of multiple cancers. Patients with metabolic syndrome and chronic inflammation show an increased risk of developing breast, endometrial, colorectal, and liver cancers [8]. People with MetS are also more prone to develop non-arteritic anterior ischemic optic neuropathy—the most vital associations were with elevated triglyceride levels, low HDL cholesterol, and high blood glucose [9]. MetS is associated with a smaller arteriolar diameter, largely driven by hypertension and elevated waist circumference [10]. MetS components can be altered through lifestyle changes, such as adopting a healthier diet and increasing physical activity. Beginning an exercise program with moderate-intensity continuous training followed by or combined with high-intensity interval training (HIIT) 3 times a week for 16 weeks on an exercise training device can help prevent, modify, or address the worsening of metabolic syndrome components [11]. According to Khan et al., a diet with higher inflammatory potential, indicated by elevated Dietary Inflammatory Index (DII) scores, is linked to an increased risk of developing metabolic syndrome in women over time. The DII scores were strongly linked to each of the five components of metabolic syndrome in women [12]. By lowering blood pressure, improving blood sugar levels and managing cholesterol individuals may also reduce oxidative stress and inflammation that contribute to eye problems. Therefore, understanding how metabolic syndrome is related to eye diseases is crucial for protecting sight and preventing vision loss associated with metabolic disorders. Another important lifestyle modification should be cutting down on drinking alcohol and smoking. Jamali et al. proved that the likelihood of MetS and elevated triglycerides was higher among individuals who consumed alcohol in the past 12 months compared to non-drinkers. Similarly, those who smoked tobacco in the past year had increased odds of having a high waist circumference compared to non-smokers among Iranian population [13]. An inverse correlation was noted between increasing altitude and MetS prevalence. At altitudes above 1500 m, the prevalence of metabolic syndrome was 36.5%, with a higher occurrence in women (35.5%). Among its components, abdominal obesity and low HDL were observed in over 40% of cases, whereas high blood pressure, elevated triglycerides, and impaired glucose levels were present in less than 30% [14].

## 2. Materials and Methods

The central aim of this study was to assess the relationship between metabolic syndrome and any form of eye disease or disorder. In order to achieve that we used reputable online databases, such as Pubmed, Web of Science and Google Scholar. We used various combinations of the following terms: “metabolic syndrome” and “inflammation”, “eye”, “eye diseases”, “glaucoma”, “cataracts”, “age-related macular degeneration”, “diabetic retinopathy”, “dry eye”, “retinal vein occlusion”, “ocular motor nerve palsy”. Our investigation was conducted in August 2024 and updated with studies published up to December 2024. Data extraction involved two independent authors, who gathered information on country, first author, publication year, study population details, intervention, study design, and follow-up duration. The summaries of articles were assessed for information relevant to the focus of our analysis. Abstracts not written in English were not included. Publications available solely as abstracts or conference posters were excluded as well. Full-text articles related to the topic were chosen after reviewing the abstracts. Results were categorized based on the specific components of metabolic syndrome and their associated ocular diseases. Any discrepancies were resolved through discussions to reach a consensus. Ultimately, we selected 90 articles to include in this review.

## 3. Inflammation as a Core Mechanism in Metabolic Syndrome

**Figure 2:**
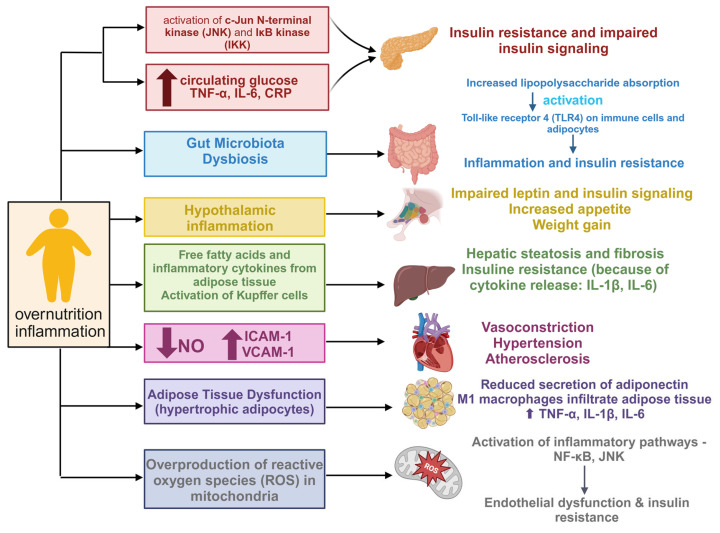
Pathways underlying MetS and inflammation. Created in BioRender. Pieńczykowska, K. (accessed on 20 February 2025) ↓ NO—the level of nitric oxide lowers, ↑ ICAM-1—intercellular adhesion molecule 1 (its level elevates), ↑ VCAM-1—vascular cell adhesion molecule 1 (its level elevates).

**Figure 3:**
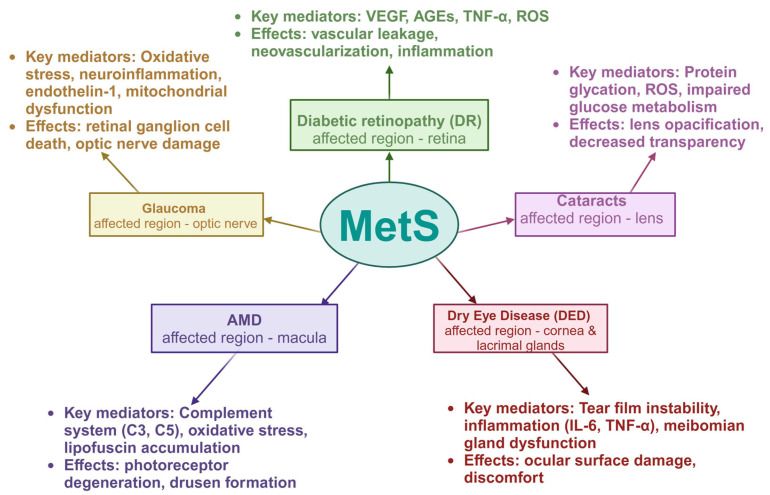
A presentation of connections between metabolic syndrome ocular disorders, highlighting the key cellular and molecular mediators and the area of the eye affected. Created in BioRender. Pieńczykowska, K. (accessed on 20 February 2025).

The development of MetS involves a combination of genetic and acquired factors, all linked to insulin resistance and persistent low-level inflammation ([15], Figure 2). Adipose tissue, particularly visceral fat, functions as an endocrine organ by secreting various pro-inflammatory cytokines, known as adipokines, such as leptin and chemerin. These adipokines contribute to systemic inflammation and are implicated in the pathogenesis of insulin resistance [16]. The diverse pathogenic mechanisms driving the progression of metabolic syndrome converge to create a pro-inflammatory state, which accounts for the elevated levels of inflammatory markers like Interleukin 6 (IL-6), C-reactive protein (CRP), and Tumor Necrosis Factor Alpha (TNFα) observed in individuals with MetS [17]. Osorio-Conles et al. have found that in subcutaneous adipose tissue from individuals with metabolic syndrome, mRNA levels of markers for M1 macrophages (CD80), M2 macrophages (MRC1/CD206), and total macrophages (CD68) were upregulated 3-, 1.35-, and 1.47-fold, respectively, compared to those without MetS. In visceral adipose tissue (VAT), only the M2 marker MSR1/CD204 showed a slight 1.28-fold increase. Hypoxia-inducible factor 1-alpha (HIF1A) and adiponectin (ADIPOQ) expressions in subcutaneous adipose tissue (SAT) were elevated 1.52- and 1.39-fold, while leptin receptor (LEPR) expression was reduced 0.62-fold. Additionally, the adipogenic marker peroxisome proliferator activated receptor alpha (PPARA) was 1.25-fold upregulated in SAT, and fatty acid binding protein 4 (FABP4) was 0.53-fold downregulated in the VAT of MetS+ individuals [18]. The strong connection between inflammation and metabolic changes in MetS particularly involves metabolites like amines, amino acids, and lipids. As for biogenic amines, elevated levels of compounds like choline, l-carnitine, and trimethylamine-N-oxide are associated with inflammation and increased metabolic dysfunction risk [19]. Branched-chain amino acids (BCAAs) such as isoleucine, leucine, and valine are linked to inflammatory markers and cardiometabolic issues in MetS. Increased isoleucine and tyrosine, along with reduced lysine and methionine, are early biomarkers of MetS. Low lysine is particularly tied to increased inflammation and blood glucose, suggesting that dietary lysine may have anti-inflammatory benefits [20]. Reduced taurine levels were linked to an increased risk of developing metabolic syndrome. This amino acid effectively reduces total cholesterol, TG, blood glucose, and blood pressure, therefore it can play a role in prevention of MetS [21]. Supplementing with taurine amplifies its plasma levels, decreases inflammatory and oxidative markers in the blood, and boosts plasma adiponectin levels in humans. Studies have shown that taurine combats obesity primarily by enhancing energy expenditure through the upregulation of factors involved in fatty acid oxidation. Additionally, it helps prevent hypercholesterolemia by facilitating the conversion of cholesterol to bile acids, increasing bile acid excretion in feces, and inhibiting bile acid reabsorption in the ileum [22]. Elevated Gamma-Aminobutyric Acid (GABA) and d-pyroglutamic acid levels are linked to inflammation in MetS. Reduced N-acetyl-d-tryptophan levels are inversely related to inflammatory markers, marking it as a significant biomarker for further study [23]. Phosphatidylcholine is the predominant phospholipid found in the membranes of all mammalian cells—essential for the assembly and stability of lipoproteins. Alongside cholesterol, phospholipids (especially phosphatidylcholine) create a monolayer that encloses a neutral lipid core composed of triacylglycerols (TAG) and cholesteryl esters [24]. Phosphatidylcholine 34:2 (PC 34:2) levels are elevated in nascent MetS and correlate with inflammatory markers, making it a potential early biomarker. PC (34:2) showed a strong positive association with waist circumference, plasma glucose, free fatty acids, and triglyceride levels. It was also significantly correlated with pro-inflammatory markers, including plasma high-sensitivity C-reactive protein (hs-CRP), Interleukin-1 beta (IL-1β) and Interleukin 8 (IL-8) [25]. Oxidative stress occurs when there is an imbalance between oxidative and antioxidant systems, leading to excessive production of reactive molecules like reactive oxygen species (ROS) and reactive nitrogen species (RNS). This imbalance results in cellular and tissue damage and is a key factor in aging and disease. ROS, primarily produced by leukocytes such as neutrophils and macrophages, play a significant role in metabolic and signaling pathways, including p38 mitogen-activated protein kinases (P38MAPK), 5′ AMP-activated protein kinase (AMPK), and Nrf2-Keap1 pathway. Transcription factors like Nuclear factor erythroid 2-related factor 2 (NRF2) and Nuclear factor-kappa B (NF-κB) regulate antioxidant responses, immune function, and inflammation, linking oxidative stress to various diseases, including inflammatory, autoimmune, and neurodegenerative disorders. In the eye, exposure to environmental factors like sunlight can trigger oxidative damage, contributing to conditions such as DNA damage and photoaging [26]. Various ophthalmic diseases, such as age-related macular degeneration, glaucoma, corneal disease, and diabetic retinopathy, are closely linked to oxidative stress. In these conditions, oxidative stress results in excessive ROS production, which can cause cellular damage, inflammation, and apoptosis, ultimately leading to structural and functional impairments in ocular tissues. Moreover, oxidative stress contributes to abnormal neovascularization, further worsening the progression of ocular diseases [27]. Oxidative metabolism in eukaryotic cells primarily occurs in mitochondria, where cellular respiration generates Adenosine triphosphate (ATP) through oxidative phosphorylation. Mitochondria are key sources of ROS and are exposed to RNS. ROS are also produced in peroxisomes, the endoplasmic reticulum, and by enzymes such as xanthine oxidase, endothelial oxidases, and NADPH oxidase (NOX). NOX enzymes facilitate electron transfer, reducing oxygen to superoxide anion. The pentose phosphate pathway (PPP), initiated by glucose-6-phosphate dehydrogenase (G6PD), generates nicotinamide adenine dinucleotide phosphate (NADPH), essential for reducing oxidative stress. UV exposure increases corneal G6PD activity, enhancing antioxidant defense. Transketolase (TKT) regulates metabolite flow in the PPP, supporting NADPH production and nucleotide synthesis [28]. The summary of the key cellular and molecular mediators and the area of the eye affected by diseases related to MetS can be found in Figure 3.

## 4. Metabolic Syndrome and Dry Eye Syndrome (DES)

Oxidative stress markers, particularly lipid peroxide, myeloperoxidase, nitric oxide synthase 3, xanthine oxidase/oxidoreductase, 4-hydroxy-2-nonenal, malondialdehyde, and reactive oxygen species tend to be significantly elevated in DES compared to healthy controls. Additionally, the redox homeostasis disruption in DES affects two key components of the ocular surface: the tear film and the conjunctiva [26]. Taking into account the factors of MetS, hyperglycemia, hyperlipidemia and hypertension are significantly linked to a higher risk of DES, whereas obesity is not [29]. The ocular surfaces of patients with metabolic syndrome have been found to display localized inflammatory abnormalities due to oxidative stress and ongoing low-grade systemic inflammation, leading to a higher prevalence of dry eye in these individuals. Patients with metabolic syndrome tend to show tear hyperosmolarity and dysfunction of the tear film—tear osmolarity values and OSDI scores were significantly higher, whereas the Schirmer test values and tear break-up time (TBUT) were significantly lower in the group of those suffering from MetS compared to the healthy group [30]. MetS affects the volume of tear secretion—the rate of lacrimal gland hypofunction in the MetS group was nearly double that of the non-MetS group and the volume of tear secretion was notably lower in the MetS group compared to both the non-MetS and pre-MetS groups [31]. In particular, women with metabolic syndrome (MetS) exhibit higher tear osmolarities, which interfere with the normal functioning of the ocular surface and lead to inflammation [32]. High triglyceride levels, as a component of MetS, could be associated with DES in female patients. This association remained unchanged even after adjusting for age, household income, education, residence, smoking, alcohol consumption, and exercise frequency. This might occur due to the fact that individuals with DES experience either aqueous-deficient or evaporative tear deficiency on the ocular surface. Inadequate tear production was linked to an excess of meibum lipid profiles, including triglycerides, which worsen DES symptoms [33]. According to Choi et al., dyslipidemia might be linked to the prevalence of dry eye syndrome in men, but not in women [34]. A history of using lipid-lowering medications was positively associated with a remarkably higher risk of developing DES by Li et al. [35]. In another case–control study by Aldaas et al., people with cholesterol levels over 200 experienced a 60% increased likelihood, while patients with triglycerides (TG) over 150, HDL levels below 40, or Low-density Lipoprotein (LDL) levels above 130 had a 40–50% greater chance of being diagnosed with DES [36]. As DES is a disease characterized by inflammation, systemic inflammation index ratios such as: neutrophil-to-lymphocyte ratio, platelet-to-lymphocyte ratio, monocyte-to-lymphocyte ratio, and neutrophil-to-lymphocyte and platelet ratio may serve as inflammatory biomarkers for predicting the risk of dry eye disease in patients with dyslipidemia [37]. Meibomian gland dysfunction (MGD) results in alterations to both the quality and quantity of meibum, which can lead to evaporative dry eye and disruption of the ocular surface, causing dry eye symptoms in certain individuals [38]. Young and middle-aged patients (aged between 18 and 54) with MGD who have no history of hypercholesterolemia may exhibit higher blood cholesterol levels compared to age-matched controls without MGD [39]. Conversely, Mussi et al. concluded that risk factors for metabolic syndrome are not distinctly linked to an increase in MGD when compared to individuals with dry eye who do not have MGD. Another interesting finding of this study is that being male is a risk factor for MGD compared to non-MGD dry eye [40].

## 5. Metabolic Syndrome and Diabetic Retinopathy (DR)

Metabolic syndrome has been proven to be a major risk factor for the onset of microvascular complications in individuals with diabetes in addition to hyperglycemia and the disease duration. Diabetic individuals with MetS experienced a significantly higher incidence of microvascular complications such as: microalbuminuria, neuropathy, retinopathy, and leg ulcers compared to those without the syndrome, with rates of 46.6% and 26.8%, respectively [41]. Patients with MetS show a higher prevalence of retinopathy, including proliferative diabetic retinopathy—the frequency of retinopathy was 9.64% among patients with MetS, compared to 3.91% in those without MetS. Elevated diastolic blood pressure independently contributes to the risk of retinopathy in people suffering from MetS [42]. Similar conclusions were drawn by Huang et al., who discovered that among the group of adults with type 1 diabetes mellitus the prevalence DR in patients with MetS was roughly twice as high as in those without MetS [43]. Karaca et al. revealed that patients with MetS showed a thinning of the inner retinal layers and photoreceptor layer in Optical Coherence Tomography (OCT) segmentation analysis. This finding suggests that intrinsic factors of MetS—such as insulin resistance and inflammation originating from adipose tissue—may contribute to neurodegeneration independently of the high blood sugar levels typically linked to diabetes mellitus [44]. The most important components of MetS in the connection between these two diseases are elevated fasting glucose and systolic blood pressure [45]. In a cross-sectional study by Lin et al., female patients with metabolic syndrome exhibited a higher prevalence of diabetic retinopathy. Additionally, patients generally had thinner central retinal arterioles as the number of metabolic syndrome components increased [46]. Osteopontin (OPN) levels in the vitreous fluid tend to be significantly higher in patients with diabetic retinopathy compared to the control group [47]. OPN might become helpful in preventing the onset of retinopathy or other retinal damage associated with the progression of MetS, since it acts as a mediator of inflammation at retinal injury sites. Over time, retinal changes emerge, including early breakdown of the blood–retinal barrier and increased OPN expression. Therefore it should be considered as an early biomarker of diabetic retinopathy among patients with MetS [48]. The inclusion of fenofibrate with statins in the group of patients with pre-existing DR was helpful in reaching a significantly reduced risk of progression of diabetic retinopathy compared to statin therapy alone in patients with type 2 diabetes and metabolic syndrome [49]. Diabetic macular edema (DME) is often a consequence of diabetic retinopathy, representing a specific manifestation of retinal damage in patients with diabetes. No link was found between the development of DME and body mass index (BMI), triglyceride levels, or high-density lipoprotein levels. An increase of 10 mm Hg in systolic blood pressure was associated with a 6% higher risk of developing DME [50]. Research on this topic remains very limited.

## 6. Metabolic Syndrome and Myopia

Myopic individuals are at a higher risk (0.82 vs. 0.21%) of developing metabolic syndrome, though the degree of myopia has minimal impact on future MetS risk. This relationship may exist because of several mechanisms. Both myopia and MetS share environmental risk factors, such as reduced outdoor and physical activity, which are associated with higher screen time and sedentary behavior, particularly in young myopic individuals. Lower levels of physical activity, common among adults with myopia, are associated with a higher MetS risk, suggesting that activity patterns in myopic individuals may contribute to MetS later in life. Additionally, shared biological pathways, including vascular inflammation and insulin signaling, connect the pathogenesis of myopia with obesity, diabetes, and MetS. Insulin and insulin-like growth factor-1 (IGF-1) are implicated in both myopia progression and MetS, providing a potential biological basis for their association [51]. Myopic retinopathy is tied to a narrowing of the retinal blood vessels. This relationship seems to be independent of the degree of myopic refraction [52]. Cheung et al. discovered a link between wider retinal venular caliber and higher BMI and body weight in children. Children with a BMI that is 3.1 kg/m^2^ higher tend to have, on average, a retinal venular caliber that is 2.55 μm wider. Inversely, BMI showed no association with retinal arteriolar caliber [53]. Further research is needed to examine the connection between MetS and myopia more precisely.

## 7. Metabolic Syndrome and Cataracts

Metabolic syndrome was strongly linked to age-related cataracts, particularly cortical, nuclear, and any type of cataract in general. The risk of developing any cataract was a 46% greater for men suffering from MetS and 49% greater for women with MetS [54]. Participants with MetS showed a higher prevalence of cataracts compared to those without MetS. Additionally, when analyzing individual components of MetS, low HDL cholesterol levels and high blood sugar were linked to an increased risk of cataracts. However, there was no significant association between abdominal obesity and cataracts [55]. Elevated glucose levels and obesity were predictors of a higher incidence of cortical cataracts over five years. Furthermore, low levels of high-density lipoprotein and high glucose were linked to a greater incidence of cortical and posterior subcapsular (PSC) cataracts over ten years, respectively [56]. MetS, characterized by abdominal obesity, diabetes, and hypertension, was also linked to a twice higher risk of cataract extraction in men aged 65 years or younger [57]. The possible mechanism of the increased risk of cataracts in patients with MetS is through the activation of the sorbitol pathway. According to Reddy et al., sorbitol levels in cataractous lenses above 650 nmoles may lead to cataracts [58]. There is also a potential genetic connection. The exploratory study made by Mehra et al. indicates that having at least one longer allele of the D2S439 marker near the *SPHKAP* gene is linked to both cataracts and components of metabolic syndrome in Asian Indians, revealing a potential shared genetic locus. The same study also suggests the presence of an additional candidate gene in the *2q* region, that is related to cataracts [59]. Among the four features of metabolic syndrome, hyperglycemia is the most significant risk factor for the prevalence of age-related cataracts, as the odds ratios for hemoglobin A1c (HbA1c) exceed 2 [60]. Females with elevated blood pressure and metabolic syndrome had a higher risk of developing cataracts compared to males [61]. Oppositely, in Korean people aged 40 and older, the rates of cataracts turned out to be similar for both genders [62]. However, when it comes to age-related cataracts, the association between this type and MetS exists specifically in Korean women, particularly in cases of nuclear cataract [63]. Metabolic dysfunction-associated fatty liver disease (MAFLD) is strongly associated with metabolic syndrome—MAFLD is considered the liver manifestation of metabolic syndrome, meaning that the same metabolic disturbances driving metabolic syndrome also lead to fat buildup and dysfunction in the liver. The metabolic and inflammatory factors associated with MAFLD may play a role in the development of cataracts. On the other hand, nonalcoholic FLD showed no association with any type of cataracts [64]. Metabolic syndrome is also regarded as a predisposing factor for severe postoperative complications of cataract surgery. Hypertension can contribute to poor postoperative outcomes, such as delayed wound healing in root canal treatments and an increased risk of postoperative endophthalmitis. Dyslipidemia can harm the vascular endothelium, leading to atherosclerotic plaque formation. The resulting weakened ocular vasculature from these dyslipidemia-associated conditions may increase the likelihood of infection, inflammation, and hemorrhagic complications [65].

## 8. Metabolic Syndrome and Glaucoma

Participants of the Korea National Health and Nutrition Examination Survey with metabolic syndrome had a significantly higher intraocular pressure (IOP) compared to those without metabolic syndrome, with each component of metabolic syndrome affecting IOP differently. The prevalence of ocular hypertension was greater in the MetS group for both men and women: 1.3% compared to 0.5% in men, and 0.7% versus 0.2% in women. Hypertension emerged as the strongest predictor of elevated IOP [66]. Serum lipid characteristics, especially HDL and triglycerides, were identified by Kang et al. as independent risk factors for normal-tension glaucoma regardless of metabolic conditions like obesity, hypertension, and type 2 diabetes. Elevated HDL and reduced TG levels may lower the risk of normal-tension glaucoma, highlighting these lipid levels as potential markers for its prevention and treatment strategies [67]. The retinal nerve fiber layer (RNFL) is composed of the axons from retinal ganglion cells. When the optic nerve is damaged, these cells are lost, impacting the integrity of the RNFL. In healthy eyes, localized RNFL defects are uncommon, making them highly useful, for example, for diagnosing glaucoma. In individuals without glaucoma, localized defects in the RNFL are linked to components of metabolic syndrome, such as central obesity, high blood pressure, and increased fasting glucose levels. This association suggests that coexisting MetS should be taken into account when assessing patients with RNFL defects [68]. According to Wygnanski–Jaffe et al. the increase in BMI showed a positive correlation with IOP exceeding 21 mm Hg [69]. In glaucoma patients, those with metabolic syndrome exhibit higher intraocular pressure and increased central corneal thickness compared to those without metabolic syndrome. However, after adjusting for central corneal thickness, the difference in intraocular pressure between the two groups was no longer significant. Metabolic syndrome was also linked to a diagnosis of ocular hypertension [70]. Another study conducted by Lee et al. in the Korean population portrayed the relationship between metabolic syndrome and increased risk of developing primary open-angle glaucoma (POAG), particularly normal tension glaucoma (NTG) [71]. An analysis of metabolic syndrome components revealed that participants with high blood pressure, elevated fasting glucose, high triglycerides, and abdominal obesity had significantly higher intraocular pressure levels compared to those without these risk factors [72]. Individuals with BMI over 30 kg/m^2^ were more likely to develop POAG compared to those with BMI between 18.5 and 22.9 kg/m^2^. Patients aged 65 and older with a higher number of metabolic syndrome components had an increased risk of developing POAG in comparison to the younger ones [73]. The prevalence of POAG rose with a greater number of MetS components in both the overall population and non-obese individuals. However, in obese individuals, the prevalence of POAG did not show any correlation with the number of MetS components [74]. Findings described by Asaoka et al. indicated that systolic blood pressure, diastolic blood pressure, TG, LDL cholesterol, HbA1c, and BMI were not significantly linked to changes in IOP over time. This suggests that liver and biliary dysfunction—evidenced by a decrease in aspartate aminotransferase (AST), an increase in alanine aminotransferase (ALT), and elevated gamma-glutamyl transpeptidase (γGTP) levels—may contribute to a progressive increase in IOP, independent of overt metabolic syndrome. High-density lipoprotein cholesterol (HDL-C) influences IOP independently of metabolic syndrome as well [75]. Metabolic, intraocular pressure, arterial pressure, and peripheral autonomic changes induced by a high-fructose diet can be reduced through concurrent exercise training. The ocular benefits resulting from exercise training probably arise from enhancements in peripheral autonomic function [76].

## 9. Metabolic Syndrome and Age-Related Macular Degeneration (AMD)

Metabolic syndrome and three of its components—BMI over 30 kg/m^2^, elevated glucose levels and high triglycerides—were identified by Ghaem et al. as predictors of progression to late-stage AMD in an Australian white population aged 70 years or younger. However, no evidence was found indicating that MetS or its components affect the risk of developing early-stage AMD [77]. A wild-type mouse model of metabolic syndrome that was given a “fast food” diet comprised of high fat, cholesterol and fructose-supplemented water, showed retinal changes resembling certain pathological features found in AMD. Thanks to this type of diet, the mice exhibited characteristics of metabolic syndrome, such as insulin resistance and increased levels of serum lipids and cholesterol. They demonstrated cytoplasmic vacuolation, a disruption of normal retinal pigment epithelium (RPE) architecture, and a reduction in RPE nuclei. Additionally, their Bruch’s membranes got thicker in comparison to mice fed with a standard diet [78]. In a study by Nagai et al., mice fed a high-fat diet accumulated oxidized low-density lipoprotein (ox-LDL) in macrophages via the renin–angiotensin system. These ox-LDL-loaded macrophages contributed to visual impairment in those mice and caused disruptions in the retinal pigment epithelium, which is essential for renewing photoreceptor outer segments [79]. High fructose creates conditions that promote the development of neovascularization in the retina by triggering the development of metabolic syndrome. It can also negatively impact the light sensitivity of rod photoreceptors [80].

## 10. Metabolic Syndrome and Retinal Vein Occlusion

Retinal vein occlusion (RVO) is the second most prevalent retinal vascular disease, second only to diabetic retinopathy. It involves the blockage of either the branch or central retinal vein, leading to possible vision changes and long-term complications [81]. In a study conducted by Stem at al., most individuals diagnosed with central RVO had multiple components of metabolic syndrome, which was defined as the presence of hypertension, diabetes mellitus or hyperlipidemia. Participants with only diabetes mellitus (without hypertension or hyperlipidemia) or only hyperlipidemia (without DM or hypertension) did not have an elevated risk of developing central RVO. In contrast, individuals with all three mentioned components of MetS experienced a 58% higher risk of developing central RVO compared to those without any of these conditions [82]. MetS and its components were found to be positively associated with an increased risk of retinal vein occlusion in young adults (aged between 20 and 40 years old). Participants diagnosed with MetS once had a 20% higher risk of developing RVO compared to those without MetS, while those diagnosed with MetS on four occasions during their health checkups had a 71% greater risk of RVO [83]. The most important criterion for RVO development seems to be blood pressure—adjusted hazard ratio was 1.610 [84]. A different study by Kim et al. emphasizes the importance of low blood HDL-C as an independent risk factor for RVO development. There was an inverse correlation between HDL levels and the occurrence of retinal vein occlusion, independent of obesity and hypertension. Additionally, a significant synergistic effect was found between low HDL levels and the presence of both obesity and hypertension [85].

## 11. Metabolic Syndrome and Ocular Motor Cranial Nerve Palsy

On the example of Korean population, the occurrence of ocular motor cranial nerve palsy rises with age, reaching its highest frequency between 75 and 79 years. The causes of this disease are diverse and include diabetes mellitus, tumors, trauma, central nervous system inflammation, infarction, aneurysms, and subarachnoid hemorrhage. However, vascular diseases were identified as the primary suspected cause—causing 52.7% of all the incidents mentioned in this study [86]. People with metabolic syndrome faced a 35% greater risk of developing ocular motor cranial nerve palsy (CNP) than those without MetS over an average follow-up period of 8 years. Each component of MetS was independently connected to an elevated risk of incident ocular motor CNP, moreover, the risk steadily increased with the number of MetS components present. The hazard ratio was also higher in males than females [87]. Research conducted by Kim et al. concluded that the presence of metabolic syndrome and obesity alone, without additional factors, shows no significant associations with a higher risk of ocular motor CNP. Nevertheless, the coexistence of an increased level of gamma-glutamyl transferase (GGT) turns out to be strongly related to ocular motor CNP development [88]. Serum GGT seems to be a readily accessible and reasonably effective marker for diagnosing metabolic syndrome, functioning independently of other MetS parameters. Moreover, GGT is directly correlated with elevated triglyceride levels [89]. Recovery from metabolic syndrome was linked to a lower risk of ocular motor CNP compared to having persistent, chronic MetS. The hazard ratio of ocular motor CNP in the chronic MetS group was 1.424 and in the group of people who recovered from MetS it was 1.168 [90]. Additional research is needed to investigate how treating metabolic syndrome impacts the onset and progression of ocular motor cranial nerve palsy.

## 12. Conclusions

**Figure 4:**
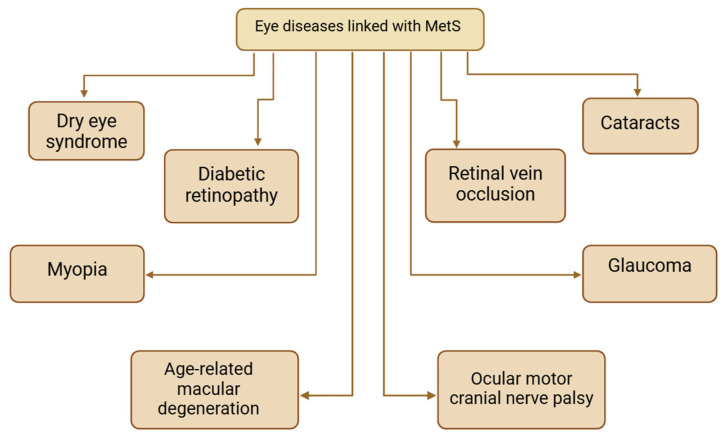
Eye diseases linked with MetS. Created in BioRender. Pieńczykowska, K. (accessed on 20 February 2025).

To wrap up, accumulating evidence highlights a significant link between metabolic syndrome and a range of ocular diseases, such as: dry eye syndrome, diabetic retinopathy, myopia, cataracts, glaucoma, age-related macular degeneration, retinal vein occlusion, and ocular motor cranial nerve palsy (as shown in Figure 4). The molecular interplay between inflammation and MetS underscores the need for targeted therapies to manage both systemic metabolic abnormalities and their ocular complications. Patients with metabolic syndrome should be considered at higher risk for these ocular conditions, accentuating the importance of regular eye exams, early diagnosis, and aggressive management of metabolic risk factors to preserve vision and prevent blindness.

## 13. Future Directions

Future research should focus on well-designed clinical trials to establish a stronger causal relationship between metabolic syndrome and ocular diseases while identifying potential therapeutic targets. Longitudinal cohort studies tracking individuals with and without MetS over time will provide critical insights into disease progression, particularly in diabetic retinopathy (DR), age-related macular degeneration (AMD), glaucoma, cataracts, and dry eye disease (DED). Interventional trials targeting MetS through lifestyle modifications, pharmacological agents (e.g., metformin, glucagon-like peptide 1 (GLP-1) agonists, anti-inflammatory drugs), or antioxidant therapy could assess their impact on ocular health, evaluating outcomes through retinal imaging, intraocular pressure, and tear film stability. Identifying how these alterations modify risk factors of eye diseases is crucial for creating public health strategies. There is a need for explanation how sex hormones interact with metabolic and ocular health and whether gender-tailored treatments are necessary. By addressing these gaps, future research can advance understanding and treatment of the complex relationship between metabolic syndrome and eye health, ultimately improving prevention, diagnosis, and management strategies for ocular complications in people with metabolic dysfunction.
